# Lean-lithium solid-state electrolytes through interfacial percolation

**DOI:** 10.1093/nsr/nwag562

**Published:** 2026-09-12

**Authors:** Jihwan Kim, Jinwoo Lee

**Affiliations:** Department of Chemical and Biomolecular Engineering, Korea Advanced Institute of Science and Technology, Republic of Korea; Department of Chemical and Biomolecular Engineering, Korea Advanced Institute of Science and Technology, Republic of Korea

Solid-state electrolytes (SSEs) are electronically insulating solids that transport Li ions between the electrodes of all-solid-state batteries (ASSBs), and their ionic conductivity is a key determinant of cell performance [1,2]. Conventionally, high conductivity has been pursued by increasing Li concentration or adopting Li-stuffed compositions, thereby increasing the number of charge carriers [3]. However, such Li enrichment increases lithium consumption and can compromise chemical and electrochemical stability [4]. Thus, there is a need for an alternative design strategy that improves the utilization, rather than simply the quantity, of mobile Li ions.

Yang and co-workers implemented an interfacial percolation strategy for oxychloride SSEs to achieve high ionic conductivity with lean Li content [5]. In this work, the authors developed a representative electrolyte, Ta₂O₅·a-LTOC (Li_1.6_Ta_2.4_O₃Cl_7.6_), consisting of Ta₂O₅ nanocrystals dispersed within a conductive amorphous Li–Ta–O–Cl matrix. The insulating Ta₂O₅ dispersoids induce Li-ion redistribution at the dispersoid–matrix interphases, producing a continuous interfacial network for rapid ion transport (Fig. 1a). As a result, Ta₂O₅·a-LTOC delivered a high ionic conductivity of 15.2 mS cm^−1^ at a lean Li content of only 1.46 wt%. When paired with a LiNi_0.9_Co_0.05_Mn_0.05_O₂ cathode, the resulting ASSBs sustained 5000 cycles at 2 C and 4000 cycles at 6 C while retaining an areal capacity above 3 mAh cm^−2^, demonstrating its practical viability.

**Figure 1.**
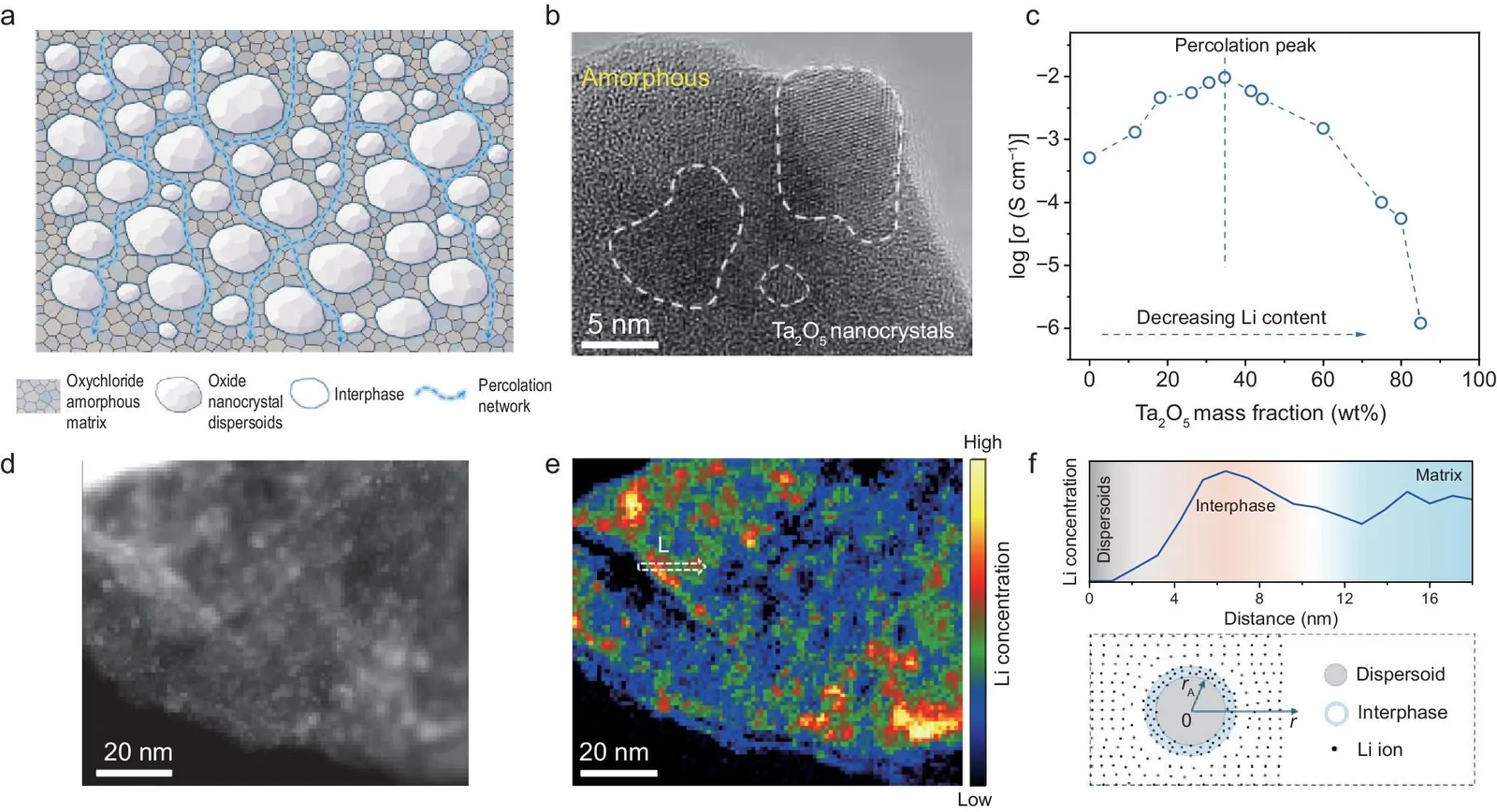
(a) Schematic diagram of percolative network. (b) Transmission electron microscopy image of Ta₂O₅·a-LTOC. (c) Ionic conductivity of solid-state electrolyte (1.63 LiCl:1.2 TaCl_5_:*k* Ta_2_O_5_) as a function of Ta_2_O_5_ mass fraction. (d) STEM image and (e) corresponding SR-EELS mapping and (f) Li concentration profile at the dispersoid interphase. Adapted with permission from Ref. [5].

The high-resolution transmission electron microscopy (TEM) image (Fig. 1b) supports this structural model, showing crystalline Ta₂O₅ domains embedded within the amorphous oxychloride matrix. The homogeneously distributed dispersoids generate an extensive interfacial area, allowing the interphases of adjacent particles to interconnect. The effect of this connectivity is reflected in the composition-dependent ionic conductivity shown in Fig. 1c. As the Ta₂O₅ mass fraction increases, the total Li content decreases, whereas the ionic conductivity increases and reaches a maximum at ∼34.7 wt%. This behavior indicates that conductivity is determined not only by the total number of Li ions but also by the connectivity of their transport pathways.

To directly probe these pathways, the authors mapped the distribution of Li in Ta₂O₅·a-LTOC using spatially resolved electron energy loss spectroscopy (SR-EELS). The scanning transmission electron microscopy (STEM) image and its corresponding SR-EELS mapping result reveal a continuous high-concentration pathway along the dispersoid–matrix interphase (Fig. 1d and e). The line profile further shows that the Li concentration increases from the Ta₂O₅ dispersoid toward the surrounding interphase and reaches a maximum within the interfacial region (Fig. 1f). These observations provide evidence that the Ta₂O₅ particles create interconnected, Li-enriched interphases that serve as pathways for rapid ion transport.

In summary, this paper demonstrates that an interfacial percolation strategy enables SSEs to achieve both low Li content and high ionic conductivity, paving the way for the deployment of lean-lithium, highly conductive SSEs for advanced ASSBs.
